# Time to achieve remission determines time to be in remission

**DOI:** 10.1186/ar3027

**Published:** 2010-05-20

**Authors:** Lydia G Schipper, Jaap Fransen, Alfons A den Broeder, Piet LCM Van Riel

**Affiliations:** 1 Department of Rheumatology, Radboud University Nijmegen Medical Centre, Geert Grooteplein 8, Nijmegen, 6500 HB, The Netherlands; 2 Department of Rheumatology, Sint Maartenskliniek Nijmegen, Hengstdal 3, Nijmegen, 6522 JV, The Netherlands

## Abstract

**Introduction:**

Though remission is currently a treatment goal in patients with rheumatoid arthritis (RA), the number of patients who achieve and sustain remission in daily practice is still small. It is suggested that early remission will be associated with sustainability of remission. The aim was to study the association between time-to-remission and sustainability of remission in a cohort of early RA patients treated according to daily practice.

**Methods:**

For this study, three-year follow-up data were used from the Nijmegen RA Inception Cohort of patients included between 1985 and 2005 (N = 753). Patients were included upon diagnosis (ACR criteria), were systematically evaluated at three-monthly visits and treated according to daily practice. Remission was defined according to the Disease Activity Score (DAS) <1.6 and the ACR remission criteria. Remission of at least 6 months duration was regarded as sustained remission. Predictors for time-to-remission were identified by Cox-regression analyses. The relation between time-to-remission and sustained remission was analyzed using longitudinal binary regression.

**Results:**

N = 398 (52%) patients achieved remission with a median time-to-remission of 12 months. Male gender, younger age and low DAS at baseline were predictive to reach remission rapidly. There were n = 142 (36%) patients experiencing sustained remission, which was determined by a shorter time-to-remission only. The relationship between time-to-remission and sustained remission was described by a significant odds ratio (1.11) (1.10 to 1.12-95% CI) that was constant over the whole period 1985 to 2005. Results obtained with the ACR remission criteria were similar.

**Conclusions:**

A shorter time-to-remission is related to sustainability of remission, supporting striving for early remission in patients with RA.

## Introduction

Expectations considering the treatment effect of rheumatoid arthritis (RA) have changed and aiming for clinical remission is currently regarded as an appropriate treatment goal in patients with early RA[1]. However, the number of patients who achieve remission in routine care is small and only a minority of these patients reach sustained remission [2,3]. Rather than complete remission, it is a near-remission disease state that currently is an achievable treatment goal in daily practice. Forthcoming treatment approaches will make the remission aim more realistic.

Starting treatment as early as possible after the diagnosis of RA is essential to provide the best clinical outcome[4]. Moreover, starting methotrexate (MTX) in combination with corticosteroids has been shown to be very successful in aiming for remission; 30 to 40% of early RA patients will experience a sustained good clinical response to MTX monotherapy [5,6]. In case MTX therapy fails, biological therapy should be added to disease-modifying anti-rheumatic drug (DMARD) therapy [5-8]. Additionally to this add-on strategy, applying tight control increases the ability to induce remission in early RA[9]. Tight control includes regular adaptations of treatment guided by the level of disease activity, i.e. remission[10]. Application of tight control may even be more important than the initial treatment given [5,9].

Following the concept of 'a window of opportunity'-successful disease course modification Is determined by aggressive treatment early in the disease course of RA - it can be hypothesized that early remission will be associated with sustainability of remission. There currently are no studies that investigated the relationship between time-to-remission and sustainability of remission. However, there are sufficient indications that in RA indeed early response is predictive for later results [11-13]. Insight into the factors that determine sustained remission early in the disease course of RA is important to provide a better long-term outcome of patients with RA.

The main objective of this study was to study the association between time-to-remission and sustainability of remission during the first three years of follow-up in a cohort of patients with early RA, who were treated according to daily practice. A second aim was to identify independent predictors of time-to-remission and sustainability or remission.

## Materials and methods

### Selection of patients

Eligible patients for this study were obtained from the Nijmegen early RA inception cohort[14]. In this cohort patients were included who were at least 18 years of age, meeting the 1987 revised American College of Rheumatology (ACR) classification criteria for RA, who had a disease duration less than one year and did not use DMARDs before[15]. Patients were visiting the outpatient clinic of the rheumatology departments of the Radboud University Nijmegen or the Maartenskliniek in Nijmegen, The Netherlands. In The Netherlands, nearly all patients with RA are treated by rheumatologists working in hospitals.

All patients were regularly assessed in three-monthly visits, but treatment decisions could be made at any time according to the discretion of the treating rheumatologist. Patients were treated with conventional DMARDs and/or biologicals and also glucocorticoids and non-steroidal anti-inflammatory drugs (NSAIDs) could be used. All clinical data on patient characteristics, medication use, clinical and laboratory measures were prospectively stored in an electronic database. All patients gave their informed consent before inclusion in the inception cohort, and the responsible local medical ethics committee had approved the study protocol. Inclusion and data collection for this cohort are still ongoing.

Since we were interested in remission during three years follow-up, all patients that were enrolled in the inception cohort between 1 July 1986 and 31 December 2005 were selected for this study.

### Clinical assessments

The following baseline patient variables were retrieved from the database: age, gender, duration of RA, rheumatoid factor positivity, disease activity (disease activity score (DAS)) and physical function (Health Assessment Questionnaire, HAQ). Disease activity was assessed at baseline and every three months thereafter by trained research nurses, using tender and swollen joint counts, erythrocyte sedimentation rate (ESR; mm/h) and patient ratings. The DAS was calculated using a 44 joint count for swelling (swollen joint count, SJC), a 53 joint count graded for tenderness (tender joint count, TJC), counted in 26 joint units (Ritchie Articular Index, RAI), general health on a Visual Analogue Scale (VAS) of 100 mm, and the value for ESR measured by the Westergren method[16]. The DAS has not the same cut points as the DAS28. A DAS ≥ 2.4 is regarded as low disease activity, and a DAS >3.7 is regarded as high disease activity[17].

Other clinical variables assessed were: duration of morning stiffness expressed in minutes, patient rating for pain, patient's global assessment of disease activity and physician's global assessment of disease activity all on a VAS from 0-100, and C-reactive protein (CRP; mg/L). Use of DMARDs, biologicals, and concomitant glucocorticoids or NSAIDs was recorded during follow-up.

### Remission definitions

Remission was defined according to a DAS < 1.6 (DAS remission) and to modified ACR remission criteria (mACR remission)[18]. Fulfillment of the mACR remission criteria required four of the following five criteria to be met: 1) morning stiffness ≤ 15 minutes, 2) VAS pain ≤ 10 mm, 3) no tender joints (out of 53 joints), 4) no swollen joints (out of 44 joints), and 5) ESR < 30 mm/h (female) or < 20 mm/h (male)[18]. In comparison with the original ACR remission criteria[19], fatigue was omitted since this item was not assessed in the cohort. Since there were three-monthly visits in our inception cohort, duration of mACR remission had to be at least three months, which differs from the duration of two months as defined in the original ACR remission criteria[19].

Patients were regarded to be in sustained remission when they maintained remission for six consecutive months, which is three consecutive visits for DAS remission and two consecutive visits for mACR remission.

### Statistical analysis

Time-to-remission was described using a Kaplan-Meier curve. A Cox proportional hazard model with time-to-remission as the dependent variable was used to calculate the hazard for achieving remission within three years for baseline variables. Variables univariately showing a significance level of p < 0.05 were included into a multivariate Cox model. The full multivariate model was reduced by stepwise removal of baseline variables with a significance level of p < 0.05.

For predicting sustained remission, a logistic regression model with achieving sustained remission as dependent variable was used to identify baseline predictors. The same variable selection procedure was followed as described above.

The relationship between time-to-remission and sustainability of remission was analyzed using longitudinal binary regression (mixed models), correcting for repeated measurements (autoregressive covariance structure) and using a logit link function for binary data. Remission during three years was the dependent variable with time-to-remission as the main covariate. The value of the DAS in the previous visit was included in the model. Other covariates were added to the model as confounders only if their addition leaded to a change of 10% or more in the effect. In addition, the following interaction terms were tested: gender with age, and calendar time with time-to-remission. Besides the interaction term with calendar time, also four sub-cohorts (inclusion between 1985-1990, 1991-1995, 1996-2000, 2001-2005) were defined, to analyze whether the relation between time-to-remission and sustained remission changed over calendar time.

For the relation between time-to-remission and sustained remission, medical treatment was regarded as an intermediate variable rather than a confounder. Treatment was not considered as a confounder because both time-to-remission and sustained remission are treatment effects. Treatment obviously is in the causal pathway and, therefore, it should not be treated as a confounder. Instead, it was analyzed whether the relation between time-to-remission and sustainability was different (effect modification) for patients treated using DMARDs in sequential monotherapy or as add-on therapy and also for patients using MTX or SASP as first DMARD. For descriptive purposes, treatment with DMARDs and glucocorticoids was studied at baseline and during three years for all sub-cohorts.

Regarding the three-year follow-up and definition of sustained remission (six months or more) there might have been patients who were not able to sustain their remission since they attained remission after two and half year. Therefore, a sensitivity analysis was performed with only patients who achieved first remission before two and half year and compared to using all patient.

In case of missing DAS values, the mean of the previous and following scores was used (linear intrapolation) for imputation. By means of sensitivity analysis, results of the analysis using the dataset after imputation were compared with the results using the dataset with missing values.

All analyses were performed separately for both DAS and mACR remission as outcome. Statistical analyses were carried out using SPSS version 16.0, statistical software package (Chicago, IL, USA) and using PROC GENMOD of SAS version 8.2 software (SAS Institute, Cary, NC).

## Results

### Baseline characteristics

Complete datasets with assessments of disease activity scores from baseline and a minimal follow-up of 140 weeks were available in 753 (86%) of the 873 included early RA patients. Patients, who were not included in this study, did not differ significantly or remarkably from patients who were included with respect to age, gender, rheumatoid factor positivity, disease duration, DAS, HAQ, medication use and change in DAS between baseline and six months (not shown).

Table 1 shows the baseline demographic and clinical variables of all patients included. Nearly all patients were included at moment of diagnosis as can be seen in the low disease duration. The patients had on average a high level of disease activity as shown by the mean DAS and the joint counts, and a moderate level of disability a shown by the HAQ.

**Table 1:** Demographic and baseline disease characteristics of patients (n = 753)

Variable and range of values	Patients included
**Age (mean [SD], years)**	54 [14]
**Women (n [%])**	477 [63%]
**Rheumatoid factor positive (n [%])**	578 [77%]
**Disease duration (median [IQR], weeks)**	0 [0-4]
**DAS (mean [SD])**	3.98 [1.28]
**DAS28 (mean [SD])**	5.07 [1.32]
**HAQ score (median [IQR])**	0.63 [0.25-1.19]
**ESR (median [IQR], mm/h)**	29 [16-48]
**CRP (median [IQR], mg/L)**	12 [1.7-37]
**44 swollen joint count (median [IQR])**	13 [8-18]
**53 tender joint count (median [IQR])**	10 [5-17]
**VAS pain, 0-100 (mean [SD], mm)**	44 [23]
**Patient's global assessment, 0-100 (mean [SD], mm)**	46 [24]
**VAS GH, 0-100 (mean [SD], mm)**	44 [22]
**Physicians global assessment, 0-100 (mean [SD], mm)**	34 [18]
**Morning stiffness (median [IQR], min)**	30 [0-90]

DAS = disease activity score based on 53 tender joint counts (Ritchie Articular Index) and 44 swollen joint counts; DAS28 = disease activity score based on 28 tender and swollen joint counts; HAQ = health assessment questionnaire; ESR = erythrocyte sedimentation rate; CRP = C-reactive protein; VAS = visual analogue scale.IQR = interquartile range, P25-P75; SD = standard deviation.

### Predictors for time-to-remission

**Figure 1:**
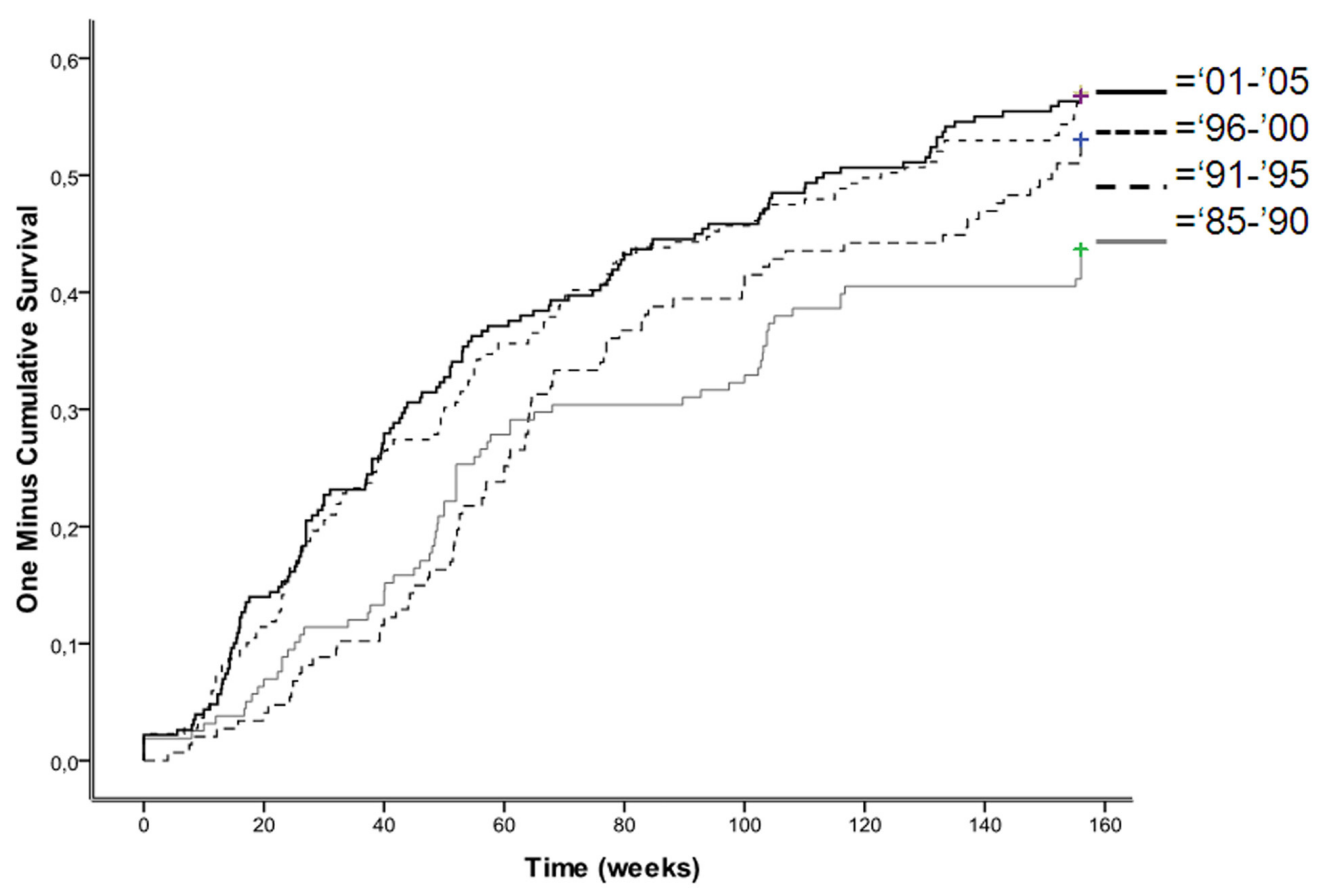
**Time to achieve DAS-remission**. Time to achieve DAS-remission within three years of follow-up in a cohort of early RA patients derived from the Nijmegen inception cohort (N = 753). Remission was defined according to the disease activity score (DAS) based on 53 tender joint counts and 44 swollen joint counts.

From all n = 753 patients, n = 398 patients (53%) achieved at least one visit in remission during the three years of follow-up. The median time-to-remission was 33 months. Figure 1 shows a Kaplan-Meier time-to-event curve of the time-to-remission for the four sub-cohorts of calendar time. The curves indicate that the earliest sub-cohort had median time-to-remission of 35 months, the following two sub-cohorts had a median time of 36 and 28 months respectively, and the last sub-cohort had a median time of 26 months to attain remission. Comparison of the early and late sub-cohorts revealed a significantly difference in this time-to-remission (P < 0.01 by overall log rank test).

Analyzing time-to-remission of only patients who attained remission within three years, resulted into a median time-to-remission of 14 months in the earliest cohort and 10 months in the latter cohort.

In Table 2 it is shown which baseline variables are univariately and multivariately predictive for time-to-remission. Univariate Cox-regression analyses showed a significant difference between the sexes: male patients reached remission sooner than female patients (18 and 36 months, respectively) (P < 0.0001). Baseline DAS was strongly predictive for time-to-remission: patients with a lower DAS at baseline achieved remission more rapidly than those with a higher DAS at baseline (P < 0.0001). A higher HAQ and higher age at disease onset were also found to prolong the time-to-remission (P < 0.01). Further, all individual components of DAS at baseline were predictive for time-to-remission (P < 0.05). The interaction between age and gender was not significant. In multivariate Cox-regression, only gender, age, and DAS were independently predictive for time-to-remission.

**Table 2:** Baseline predictors for time-to-DAS remission (n = 753)

	Univariate^1^			Multivariate^2^		
**Variable**	**Beta (β)**	**P value**	**HR (95%CI)**	**Beta (β)**	**P value**	**HR (95%CI)**

**RF positive**	0.114	0.328	1.121 (0.892-1.408)			
**Male gender**	-0.641	0.000	0.527 (0.433-0.641)	-0.511	0.000	0.600 (0.469-0.767)
**Age**	-0.009	0.008	0.991 (0.984-0.998)	-0.007	0.047	0.993 (0.986-0.999)
**Baseline DAS**	-0.364	0.000	0.695 (0.635-0.760)	-0.693	0.000	0.500 (0.434-0.576)
**Baseline HAQ**	-0.436	0.000	0.647 (0.529-0.791)			
**Dis duration**	-0.005	0.217	0.995 (0.987-1.003)			
**44 SJC**	-0.018	0.007	0.982 (0.969-0.995)			
**53 TJC**	-0.057	0.000	0.945 (0.932-0.958)			
**ESR**	-0.004	0.048	0.996 (0.992-0.999)			
**VAS GH**	-0.017	0.000	0.983 (0.978-0.987)			

^1 ^Results of univariate Cox proportional hazard models with baseline predictors for time-to-DAS remission (disease activity score, DAS<1.6) up to three years of follow-up, given with hazard ratios (HR) and 95% confidence intervals (95%CI). ^2 ^Results of the final multivariate Cox proportional hazard prediction model with independent baseline predictors for the time-to-remission (disease activity score, DAS<1.6) up to three years of follow-up, given with hazard ratios (HR) and 95% confidence intervals (95%CI).

RF = rheumatoid factor; DAS = disease activity score based on 53 tender joint counts (TJC) and 44 swollen joint counts (SJC); HAQ = health assessment questionnaire; Dis = disease; ESR = erythrocyte sedimentation rate; VAS = visual analogue scale; GH = general health. Age was measured in years and disease duration in weeks.

### Predictors for sustained remission

Of the 398 patients who ever attained remission, 142 (36%) patients had sustained remission with a median time of being in remission of 19 months. Table 3 shows the univariate and multivariate logistic regression analyses to determine baseline predictors for reaching sustained remission. Univariately, sustained remission was predicted by a shorter time-to-remission, and lower DAS and HAQ at baseline. Also, the tender joint count at baseline was predictive for sustained remission. In multivariate regression, none of the baseline variables were independently associated with sustained remission, except for time-to-remission. No significant interaction terms were detected. Thus, time-to-remission emerged as an important predictor of sustained remission.

**Table 3:** Baseline predictors of sustained DAS remission (n = 753)

	Univariate^1^			Multivariate^2^		
**Variable**	**Beta (β)**	**P value**	**OR (95% CI)**	**Beta (β)**	**P value**	**OR (95% CI)**

**Time-to-remis**	-0.091	0.000	0.913 (0.889-0.939)	-0.094	0.000	0.910 (0.878-0.944)
**RF positive**	0.447	0.058	1.563 (0.985-2.481)			
**Male gender**	-0.379	0.067	0.685 (0.457-1.026)			
**Age**	0.007	0.302	1.007 (0.993-1.022)			
**Baseline DAS**	-0.302	0.001	0.740 (0.620-0.882)			
**Baseline HAQ**	-0.567	0.011	0.567 (0.367-0.878)			
**Dis duration**	-0.003	0.745	0.997 (0.982-1.013)			
**44 SJC**	-0.012	0.384	0.988 (0.963-1.015)			
**53 TJC**	-0.070	0.000	0.932 (0.903-0.962)			
**ESR**	-0.004	0.349	0.996 (0.988-1.004)			
**VAS GH**	-0.009	0.075	0.991 (0.980-1.001)			

^1 ^Results of the univariate logistic regression model for sustaining remission (DAS<1.6 for at least 6 months or more) during 3 years of follow-up and baseline variables, given with odds ratios (OR) and 95% confidence intervals (95%CI). ^2 ^Results of the final multivariate logistic regression model for sustaining remission (DAS<1.6 for at least 6 months or more) during 3 years of follow-up and baseline variables, given with odds ratios (OR) and 95% confidence intervals (95% CI).

Remis = remission; RF = rheumatoid factor; DAS = disease activity score based on 53 tender joint counts (TJC) and 44 swollen joint counts (SJC); HAQ = health assessment questionnaire; Dis = disease; ESR = erythrocyte sedimentation rate; VAS = visual analogue scale; GH = general health. Time-to-remission was measured in months, age was measured in years and disease duration was measured in weeks.

### Relationship between time-to-remission and sustained remission

Table 4 shows the descriptives of time-to-remission and sustained remission and the relation between time-to-remission and sustained remission. The median time needed to reach remission was 12 months. The median time that remission sustained was 19 months. The odds ratio (OR) (95% CI) of the relation between time-to-remission and having sustained remission was 1.11 (1.10-1.12) (P < 0.0001). As time-to-remission was calculated in months, this means that patients who achieved first remission one month earlier, had a higher chance on sustained remission, an OR of 1.11 than patients who achieved first remission one month later. Achieving remission three months earlier resulted in an OR of 1.37 to remain in remission. In case of one year earlier remission, this OR even increased to 3.5 to keep sustained remission. Accordingly, the chance on sustained remission increases with every month time-to-remission is shorter. Illustratively, the median time-to-remission in patients with sustained remission was 9 months (interquartile range, IQR 4-13 months) while time-to-remission in patients with non-sustained remission was 13 months (IQR 7-24 months) (P < 0.0001). There were no baseline variables (such as age and gender) that acted as confounders, only the DAS value of the previous visit was included as covariate in the model.

**Table 4:** Relationship between time-to-remission and sustained remission

	All patients (n = 753)	1985-1990 (n = 147)	1991-1995 (n = 158)	1996-2000 (n = 219)	2001-2005 (n = 229)
***DAS remission***					
**Achieving remission (n [%])^1^**	398 [53%]	77 [52%]	68 [43%]	124 [57%]	129 [56%]
**Time-to-remission** **(median [IQR], months)**	33 [11-36]	35 [14-36]	36 [12-36]	28 [9-36]	26 [9-36]
**Sustained remission (n [%])^2^**	142 [36%]	22 [29%]	25 [37%]	46 [37%]	49 [38%]
**Time in sustained remission** **(median [IQR], months])**	19 [10-28]	9 [6-22]	22 [14-29]	18 [10-26]	22 [13-29]
**Relationship between time to** **achieve and sustained remission** **(OR [95%CI])^3^**	1.11 [1.10-1.12]	1.09 [1.07-1.11]	1.15 [1.12-1.17]	1.09 [1.08-1.11]	1.13 [1.11-1.15]
***mACR remission***					
**Achieving remission (n [%])**^4^	226 [30%]	48 [33%]	45 [29%]	62 [28%]	71 [31%]
**Time-to-remission** **(median [IQR], months)**	13 [8-24]	15 [10-28]	13 [7-24]	15 [9-23]	10 [7-19]
**Sustained remission (n [%])**^5^	58 [26%]	8 [17%]	18 [33%]	13 [21%]	19 [27%]
**Time in sustained remission** **(median [IQR], months])**	10 [6-16]	7 [6-8]	11 [7-16]	7 [6-16]	13 [7-24]
**Relationship between time to** **achieve and sustained remission** **(OR [95%CI])**^3^	1.15 [1.14-1.16]	1.13 [1.09-1.16]	1.12 [1.07-1.18]	1.08 [0.96-1.22]	1.12 [0.93-1.16]

^1 ^Number of patients achieving at least one period of remission (disease activity score, DAS<1.6) during 3 years follow-up. ^2 ^Number of patients who had sustained DAS remission (6 months or more) during 3 years follow-up. ^3 ^Odds ratios (ORs) of remission during 3 years follow-up analyzed by longitudinal binary regression with remission status over time as dependent variable, time-to-remission (months) and DAS value of the previous visit as main covariates. ^4 ^Number of patients achieving at least one period of remission (modified American College of Rheumatology, mACR) during 3 years follow-up. Fulfillment of the mACR remission criteria was based on 4 of the following 5 criteria to be met: 1) morning stiffness ≤ 15 minutes, 2) VAS pain ≤ 10 mm, 3) no tender joints (out of 53 joints), 4) no swollen joints (out of 44 joints), and 5) ESR < 30 mm/h (female) or < 20 mm/h (male). ^5 ^Number of patients who had sustained mACR remission (6 months or more) during 3 years follow-up. IQR = interquartile range, P25-P75; CI = confidence interval.

Sensitivity analyses with only patients who attained remission before two and half year, resulted in an OR of 1.1. Also, using the dataset with missing values did not alter the above OR.

### Sustained remission during calendar time

The cohort was divided into four sub-cohorts according to calendar time (Table 4). The number of patients who achieved remission was comparable between the first and the latter cohort. Sustained remission, on the other hand, occurred more frequently in the latter cohort. Time-to-remission was longer in the beginning of the cohort (1985-1990) and also time in remission was less in the early years of the cohort (Table 4). Despite these differences, the relationship between time-to-remission and sustained remission remained constant over calendar time as can be seen by the OR of each sub-cohort that varied from 1.09 to 1.15 with great overlap of the four confidence intervals.

### mACR remission

**Figure 2:**
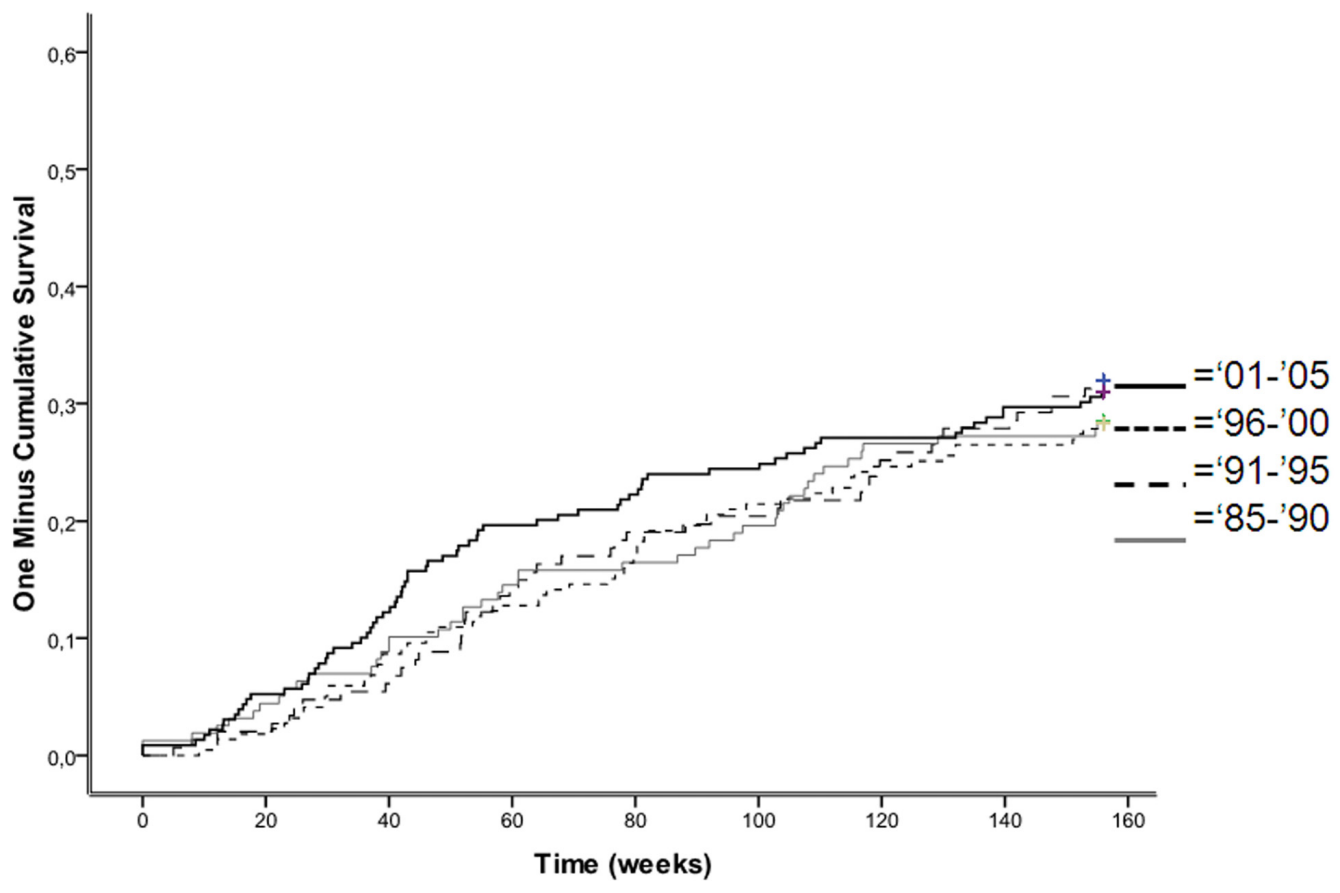
**Time to achieve mACR-remission**. Time to achieve mACR-remission within three years of follow-up in a cohort of early RA patients derived from the Nijmegen inception cohort. Remission was defined according to the modified American College of Rheumatology (mACR) criteria based on fulfilment of 4 of the 5 criteria: 1) morning stiffness ≤ 15 minutes, 2) VAS pain ≤ 10 mm, 3) no tender joints (out of 53 joints), 4) no swollen joints (out of 44 joints), and 5) ESR < 30 mm/h (female) or < 20 mm/h (male).

Overall, mACR remission occurred less frequently than DAS remission (Table 4). The independent predictors for time-to-mACR remission were comparable with those found for achieving DAS remission. Time-to-remission for the four sub-cohorts is shown in Figure 2. Again, the earlier sub-cohorts had a longer median time-to-remission than the last sub-cohort (P < 0.01). To sustain mACR remission was also more difficult than DAS remission. Time-to-remission was the strongest and single predictor of sustained mACR remission. The relationship between time-to-remission and sustained remission was again significant (OR = 1.15) and remained constant over calendar time.

### Medication use

Among the 753 patients included, 720 patients started monotherapy (14% methotrexate (MTX), 67% sulphasalazine (SASP), 15% hydroxychloroquine and 4% other DMARDs) and 33 patients received DMARDs combination therapy at baseline, mainly MTX combined with SASP. During three years, 29% patient received a combination of DMARDs (mostly MTX plus SASP) as an add-on strategy applied and 71% of the patients received a sequential strategy of DMARDs. In the beginning of the cohort, 5% of the patients were given combination therapy, which increased to 39% in the last sub-cohort. Further, in earlier cohorts SASP was in 54% the starting drug compared to 24% in the latter cohorts. The use of MTX increased from 1% to 16%. Biologicals were given to 17% of the patients. Overall, 19% of the patients used prednisone and 49% received at least one intra-muscular/intra-articular injection of prednisolone with a median of number of two (IQR 1-4).

With respect to patients who sustained their remission (n = 142), nearly all (96%) patients started with monotherapy and SASP was described as first DMARD in 69%. A higher proportion of patients received DMARDs in sequential monotherapy until their first remission (87%) compared to the whole patient group (71%) and a DMARD add-on strategy was less commonly applied (13% versus 29%). Also, the percentage of anti-TNF users before first remission was actually low (3%). Further, 11% of the patients received prednisone and 35% at leas one intra-muscular/intra-articular injection of prednisolone (median number of 1).

### Remission and medication use

Since more patients started with SASP as first-line DMARD, we investigated remission in both SASP and MTX first-line patients. Whether treatment was started with MTX or with SASP did not predict time-to-remission (p = 0.412), sustainability of remission (p = 0.091), nor did it modify the relationship between time-to-remission and sustainability of remission (p = 0.153). Further, patients in sustained remission received more often DMARDs in sequential monotherapy. Therefore, patients were stratified according to treatment strategy: DMARD sequential monotherapy (70%) or add-on therapy (30%). The relation between time-to-remission and sustainability was not different between both treatment groups (p = 0.609).

## Discussion

This study was conducted to identify predictors for achieving and sustaining remission and to investigate the relationship between time-to-remission and sustained remission according to two different remission criteria in a cohort of early RA patients treated in daily practice between 1985 and 2005. According to the results of this study, the number of patients achieving remission was comparable during the whole time frame of the cohort. Predictors to achieve more rapidly DAS remission were male gender, younger age and a low DAS or HAQ at baseline. Sustained remission was only and mainly determined by time-to-remission; the chance of sustained remission increased significantly with decreasing time-to-remission. Over time, reflecting more intensive treatment, the time-to-remission tended to shorten, the occurrence of sustained remission tended to increase, but the relation between time-to-remission and sustainability remained fairly constant. This indicates that the relation between time-to-remission and sustainability does not heavily depend on the type or strategy of DMARDs given. Results obtained with the mACR remission criteria were similar.

This study is the first daily care study showing the influence of time-to-remission at sustained remission. In earlier studies on evaluation of remission in daily practice, comparable predictors have been identified for achieving remission in patients with early RA [20,21]. Rheumatoid factor [11,20] and anti-cyclic citrullinated peptide (anti-CCP) antibody status [22], level of CRP[23] and presence of erosions at baseline[20] have also shown to be predictive for not achieving remission rapidly. Further, the early start of DMARDs combination therapy[24] or anti-TNFα agents plus MTX [5,7,8] in RA patients emerged to be predictive for sustained remission.

Since treatment in patients with RA has shifted towards a more early and aggressive treatment strategy, higher remission rates and more sustainability of remission are expected these days. Remarkably in this study, the association between time-to-remission and sustained remission was present in all cohort patients, irrespective of date of inclusion. Therefore, early remission seems to be essential for sustained remission, and thus the further course of RA. Earlier studies have already confirmed this implication. In addition, the frequency of remission after one year was significantly higher among responders than among the non-responders [11,25] and achieving low disease activity within three months of treatment was associated with low disease activity or remission at one year[12].

Several criteria of (sustained) clinical remission are available and remission results of studies may for this reason depend on the remission criterion used [3,26]. This study applied both DAS and mACR as remission criteria, which resulted in similar predictors for attaining and sustaining remission. Moreover, the relationship between time-to-remission and sustained remission remained significant. Reaching and sustaining mACR remission was only more difficult than DAS remission. Additionally, a great proportion of patients (23%) who attained DAS remission did not fulfill mACR remission. Since mACR remission criteria include absence of both tender and swollen joints, remission according to mACR is regarded as very strict[27].

For the aim of this study, we used cohort data from the Nijmegen inception cohort. Cohort data have the advantage to be closely related to daily practice care[28] and, therefore, the patients included in this study are supposed to be representative of the general RA population attending outpatient clinics. Moreover, the inception cohort from this study is regarded as a very valuable and complete cohort since this cohort includes a long time span, started from 1985 and still ongoing, and clinical variables are systematically collected every three months.

However, a limitation of using data from daily practice is that medication use differs for each patient and changes over time. For that reason, medication use cannot be analyzed as would it be an effect-modifier and studying medical treatment may be complicated using cohort data. Therefore, medication use in this study was regarded as an intermediate variable and was described for each sub-cohort to get more insight into time-trends of medication. Further, we have demonstrated that despite medication adjustments at the discretion of rheumatologists, the treatment strategy applied was mostly a sequential or step-up strategy (with or without glucocorticoids), starting with either MTX or SASP and the prescription of anti-TNF agents was low.

The number of anti-TNF users in this study was low. On the one hand the study includes the period 1990-2000 when anti-TNF was not available, on the other hand because in the Netherlands, anti-TNF is used after failure on at least two DMARDs. The results of this study, therefore, do not automatically generalize to patients treated with anti-TNF. Leaving out the patients treated with anti-TNF from the analysis did not change the results (not shown). Further research should be necessary to investigate, and even generalize, the relationship between time-to-remission and sustained remission in patients using (their first) anti-TNF treatment.

In some patients, joint damage may proceed despite clinical remission [29,30], However, low levels of inflammation and specifically remission are associated with less (further) progression of joint damage [31,32]. Clinical remission and ultimately the halt of progression of joint damage is regarded as the current treatment goal in RA[1]. In clinical trials, remission has already shown to be attainable [7,33,34] and striving for a sustained state of (drug-free) remission has become the ultimate aim in RA[35]. However, the rate of achieving and sustaining (mACR) remission in daily practice is still very low. The results of this study have shown that within three years, 53% and 30% of the patients achieved at least one visit in DAS or mACR remission, which are comparable (or even higher) to those found in other daily care studies [2,5,9,11,18,36]. A state of sustained clinical remission was in this study difficult to reach (23-36%), which was also demonstrated in previous studies [11,37].

Despite the relatively low percentage of sustained remission, there are arguments to believe that substantial increases in sustained remission rates are these days expected. Additionally, treatment strategies with conventional DMARDs can be improved considerably by applying tight control of disease activity, including a medication protocol with regular assessments of disease activity and a threshold (remission) to determine whether treatment has to be changed [9,34,38,39]. Moreover, in clinical trials the early introduction of DMARDs in combination with prednisone or anti-TNF, applied as a 'step-down' strategy [5,6], has shown to be very effective. However, in daily practice this is not a common treatment strategy. Therefore, starting anti-TNF therapy more rapidly, in DMARDs failures and patients with poor prognosis at baseline in particular, may be necessary for achieving higher remission rates.

## Conclusions

In conclusion, the results of this study show that attaining first remission sooner, chance of sustained remission is becoming significantly higher. This relationship between time-to-remission and sustained remission remained constant over the whole cohort period from 1985 to 2005. The fact that time-to-remission is the strongest predictor for sustained remission supports the fact that aiming for remission as soon as possible is the treatment goal in patients with early RA. Aiming for remission will be better achievable with treatment strategies applied as tight control. By measuring disease activity and targeting a low value in the measure (remission) we use, remission is achievable and even sustained remission. Tight control may be applied with any DMARD and all DMARDs may be needed to get remission, For many patients with RA, MTX alone, or in combination with corticosteroids, will give the desired state of sustained remission.

## Abbreviations

ACR: American College of Rheumatology; Anti-CCP: Anti-Cyclic Citrullinated Peptide; CI: Confidence Interval; CRP: C-reactive protein; DAS: Disease Activity Score; DMARDs: Disease-Modifying Anti-Rheumatic Drugs; ESR: Erythrocyte Sedimentation Rate; GH: General Health; HAQ: Health Assessment Questionnaire; HR: Hazard Ratio; IQR: InterQuartile Range; mACR: Modified American College of Rheumatology; MTX: Methotrexate; NSAIDs: Non-Steroidal Anti-Inflammatory Drugs; OR: Odds Ratio; RA: Rheumatoid Arthritis; RAI: Ritchie Articular Index; RF: Rheumatoid Factor; SASP: Sulphasalazine; SD: Standard Deviation; SJC: Swollen Joint Count; TJC: Tender Joint Count; VAS: Visual Analogue Scale.

## Competing interests

The authors declare that they have no competing interests, neither financial, nor non-financial. The work of L. Schipper is supported by a grant from Wyeth Pharmaceuticals for the implementation of a tight control strategy in daily clinical practice. Others than the authors did not influence the content of this manuscript. Wyeth Pharmaceuticals did not have any influence on the objectives, methods, results or interpretation of the results, or conclusions of this study.

## Authors' contributions

LS has made substantial contributions to conception and design of manuscript. LS has analyzed and interpreted the data. LS has been involved in drafting and writing the manuscript. JF has made substantial contributions to conception and design of manuscript. JF has contributed to interpretation of data. JF has been involved in revising the manuscript. AB has made substantial contributions to conception and design of manuscript. AB has contributed to interpretation of data. AB has been involved in revising the manuscript. PvR has made substantial contributions to conception and design of manuscript. PvR has contributed to interpretation of data. PvR has been involved in revising the manuscript. All authors read and approved the final version of the manuscript to be submitted.
